# Editorial: Deciphering the etiology of rare genetic disorders associated with protein phosphatases

**DOI:** 10.3389/fcell.2023.1241606

**Published:** 2023-08-14

**Authors:** Veerle Janssens, Brian E. Wadzinski, Chenchen Li, Richard E. Honkanen

**Affiliations:** ^1^ Laboratory of Protein Phosphorylation and Proteomics, Department of Cellular and Molecular Medicine, University of Leuven (KU Leuven), Leuven, Belgium; ^2^ KU Leuven Brain Institute (LBI), Leuven, Belgium; ^3^ KU Leuven Cancer Institute (LKI), Leuven, Belgium; ^4^ Department of Pharmacology, Vanderbilt University School of Medicine, Nashville, TN, United States; ^5^ Department of Biochemistry and Molecular Biology, University of South Alabama, Mobile, AL, United States

**Keywords:** protein phosphatase (PP), genetic disorder, PPP2R5D, developmental disorder, signal transduction, *de novo* variant, PPP2CA, PTPN11

The reversible phosphorylation of proteins controls nearly all signal transduction pathways in human cells. Therefore, it is not surprising that aberrations in this major post-translational protein regulation mechanism are linked to many forms of disease, including inborn developmental disorders. While many efforts have been directed to understand the role of protein kinase dysfunction in the etiology, progression, and treatment of disease, the role of protein phosphatases, although equally critical, has been understudied. However, advances in DNA sequencing technologies and functional genomic approaches have allowed the discovery of many novel genetic disorders caused by protein phosphatase dysfunctions, with many more likely to follow.

In higher eukaryotes, there are two major families of protein phosphatases based on their dephosphorylation specificities: serine/threonine protein phosphatases (PSP) and tyrosine phosphatases (PTP). In this Research Topic dedicated to the etiology of rare genetic disorders associated with protein phosphatases, two manuscripts review the current knowledge on inherited or *de novo* (pathogenic) germline variants in protein phosphatase encoding genes and discuss their causal relationship with specific developmental disorders. In the first review (“*Hereditable variants of classical protein tyrosine phosphatase genes: Will they prove innocent or guilty?*”), Hendriks et al. focus on a subset (37 classical members) of phosphatases from the PTP superfamily. They comprehensively summarize the reported germline variants of these genes, and discuss their known or putative functional implications and relation to inborn diseases. In the second review (“*The role of serine/threonine phosphatases in human development: Evidence from congenital disorders”*), Vaneynde et al. go through the same effort for the 38 human genes encoding enzymes with catalytic activity against phosphoserine or phosphothreonine residues (i.e., genes encoding either monomeric PSP phosphatases or genes encoding the common catalytic subunit shared by multimeric PSP phosphatases), and the >100 genes encoding a regulatory subunit of the PSPs that function within holoenzymes (e.g., B-subunits of PP2A family phosphatases). They eventually identify 19 PSP genes that, upon mutation, can convincingly be linked to the etiology of a rare developmental disorder.

Both of the first two reviews highlight the vast number of PTP and PSP genes associated with rare developmental disorders. However, with the rapid advancement of technologies aiding functional genomic approaches to explore the etiology of novel genetic disorders, many more phosphatase disease genes will likely be discovered in the near future. Lyulcheva-Bennett et al. nicely illustrate this prediction in a retrospective study of data collected by the Genomics England Research Consortium (“*A retrospective analysis of phosphatase catalytic subunit gene variants in patients with rare disorders identifies novel candidate neurodevelopmental disease genes”*). By performing a systematic survey of *de novo* variants amongst 189 genes encoding phosphatase catalytic subunits found in rare disease patients recruited to the United Kingdom 100,000 Genomes Project (100 kGP), they reveal that 49% of phosphatase genes carry *de novo* mutation(s) in this cohort. Only 25% of these phosphatases had been previously linked to genetic disorders, and at least 9 novel candidate rare-disease genes are identified: *PTPRD*, *PTPRG*, *PTPRT*, *PTPRU*, *PTPRZ1*, *MTMR3*, *GAK*, *TPTE2*, *PTPN18*. Of note, they did not include any of the phosphatase regulatory subunit encoding genes in their study, which would likely have extended the number of new phosphatase disease genes in this cohort even further.

**FIGURE 1:**
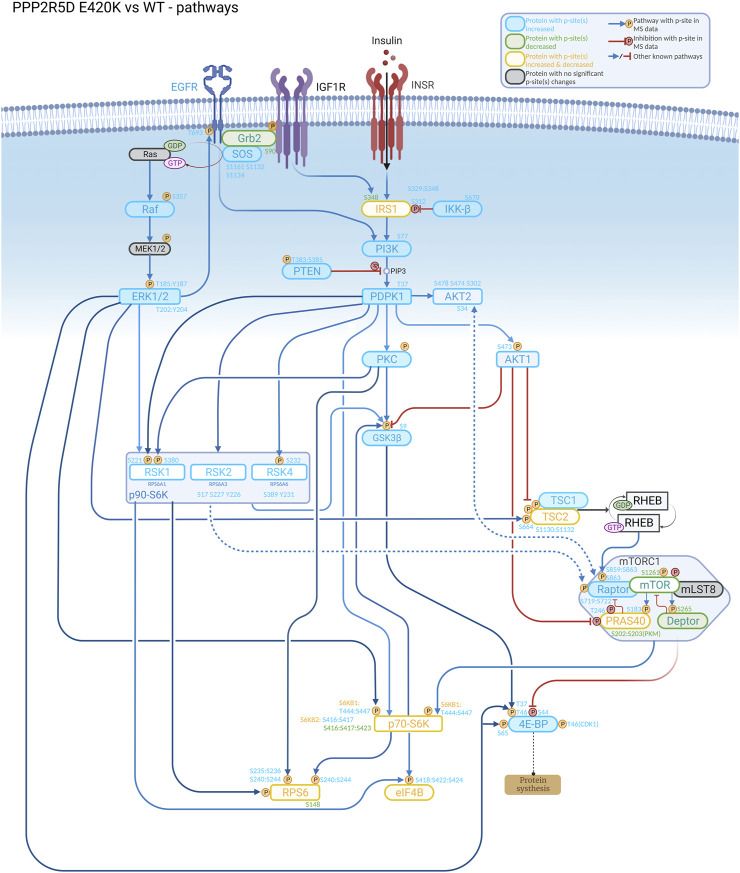
Understanding the biological role of the encoded protein is often useful to aid discovery of how a genetic variant alters normal function, as illustrated in this signaling cascade that is altered in a cell-based model of PPP2R5D-related disorders (Jordan’s syndrome). Genomic analysis revealed a single base variant in PPP2R5D resulting in the E420K pathogenic variant. To help illuminate the etiology of Jordan’s syndrome, CRISPR-single base editing was used to generate a HEK-293 cell line recapitulating a pathogenic genomic transition. Employing tandem mass tag (TMT) mass spectrometry to detect changes in phosphopeptide abundances and bioinformatic approaches to identify enrichment associated with known signaling networks, aberrant activation of mTORC1 signaling emerged from the data collected comparing wild type and variant cells, suggesting inhibitors of mTORC1 (e.g., rapamycin) may be useful to partially restore normal function to PPP2R5D E420K variant cells.

To accurately determine how rare pathogenic genomic variants alter normal biology, clinical characteristics need to be linked to altered biological processes. These characteristics may manifest at the molecular, cellular, or tissue/organ level. Thus, diverse techniques (e.g., cryogenic electron microscopy, BioID, quantitative proteomics, quantitative phosphoproteomics, and RNAseq) are required to reveal how the variant protein differs structurally and functionally from the wild-type protein, and diverse *in vivo* (e.g., mouse, *Drosophila*, *C. elegans*, zebrafish) and cellular models (e.g., CRISPR-genomic editing to recapitulate the genetic variant of interest) are used for these purposes. Often, revealing the etiology of a pathogenic variant also requires studies to first discover previously unknown biological functions (and mechanisms that regulate the functions) of the wild-type protein (Figure 1).

These aspects are all addressed in the three remaining papers of this Research Topic. Solman et al. comprehensively review the role of mutations in *PTPN11*, encoding the Src homology region 2-containing protein tyrosine phosphatase 2 (SHP2), in multiple rare hereditary diseases, such as Noonan Syndrome and Noonan Syndrome with Multiple Lentigines (“*Modeling (not so) rare developmental disorders associated with mutations in the protein-tyrosine phosphatase SHP2*”). They focus on the different model systems used to advance the understanding of the pathogenesis of these rare diseases, addressing invertebrate fruit fly models, vertebrate zebrafish and mouse models, and studies with human induced pluripotent stem cells. Verbinnen et al. illustrate the use of several *in vitro* biochemical techniques (e.g., co-immunoprecipitation, western analysis and phosphatase assays) to determine how a genetic variant may alter the normal function of the phosphatase. In their case report (“*Clinical and molecular characteristics of a novel rare de novo variant in PPP2CA in a patient with a developmental disorder, autism, and epilepsy”*) they describe a new pathogenic variant in *PPP2CA*, encoding the catalytic C*α* subunit isoform of protein phosphatase 2A (PP2A), and demonstrate how a single amino acid substitution affects PP2A activity and holoenzyme assembly. PP2A phosphatases are amongst the more recently discovered players in rare developmental disorders, and different PP2A subunit encoding genes can be involved. Most notably, *PPP2R5D*, encoding the PP2A regulatory B56δ subunit, seems to be most frequently affected, with the large majority of mutations affecting a conserved acidic loop that is in direct contact with the catalytic subunit. Extending previous studies of PPP5C, Salter et al. perform modeling studies that reveal a conserved arginine residue in PPP2CA (Arg89) that is involved in catalysis, interacts with Glu198 in PPP2R5D via an electrostatic interaction to suppress catalytic activity (“*Quantum-based modeling implies that bidentate Arg89-substrate binding enhances Serine/Threonine Protein Phosphatase-2A (PPP2R5D/PPP2R1A/PPP2CA)-mediated dephosphoryla*t*ion”*). Since the p.E198K variant is a recurrent variant in patients with the *PPP2R5D*-related disorder/Jordan’s Syndrome, their quantum-based modeling indicates that this substitution may directly affect PP2A activity in the patients.

We hope that this Research Topic, unique because of its specific focus on protein phosphatases, will increase interest in the role of these important enzymes in genetic diseases and encourage more studies that dig deeper into missing genotype-phenotype correlations and pathogenic mechanisms. In addition, studies with genetic variants may also help shed light on the role of phosphatases in normal biological processes, which will in turn may inform roles of phosphatases in the pathophysiology of metabolic disorders and cancers. A better understanding of the downstream targets and effectors of the affected phosphatases should eventually provide the much-needed perspectives for the development of therapeutic interventions and treatment options for patients with these rare disorders.

